# Association between atherogenic index of plasma and post-stroke epilepsy within one year in patients with acute ischemic stroke: a retrospective cohort study

**DOI:** 10.3389/fneur.2026.1794488

**Published:** 2026-03-30

**Authors:** Ziying Wang, Lingling Wang

**Affiliations:** 1 Department of Postgraduate, School of Clinical Medicine, Beihua University, Jilin, China; 2 Stroke Unit, Affiliated Hospital of Beihua University, Jilin, China

**Keywords:** acute ischemic stroke, atherogenic index of plasma, C-reactive protein, hemoglobin A1c, National Institutes of Health Stroke scale, post-stroke epilepsy

## Abstract

**Introduction:**

Acute ischemic stroke (AIS) is a major cause of death and disability globally. Post-stroke epilepsy (PSE) adversely affects prognosis and quality of life. This retrospective observational study was to explore the association between the atherogenic index of plasma (AIP) and PSE within one year after AIS.

**Methods:**

This study was a retrospective observational cohort analysis of 20,538 middle-aged and elderly patients with AIS. AIP was calculated as log_10_ (TG/HDL). Participants were stratified by AIP levels. Multivariable logistic regression, restricted cubic spline analysis, mediation, stratified and interaction analysis were performed.

**Results:**

Elevated AIP was significantly associated with higher odds of PSE. A non-linear threshold effect was identified, with an overall inflection point at 0.048 (males: 0.049; females: 0.026), and the association between AIP and PSE was stronger below this threshold. Mediation analysis indicated a reciprocal mediation between fibrinogen and AIP on PSE. Significant multiplicative and additive interactions of AIP with sex, National Institutes of Health Stroke Scale (NIHSS) and hemoglobin A1c (HbA1c) were observed. Significant additive interactions of AIP with C-reactive protein (CRP) were observed.

**Conclusion:**

Elevated AIP was associated with higher risk of PSE within one year after AIS. This association exhibited sex-specific threshold effects, a bidirectional mediating relationship with fibrinogen, and interactive effects with sex, HbA1c, CRP, and NIHSS. As a simple metric derived from routine lipid profiles, AIP may be useful for risk stratification.

## 1 Introduction

Acute ischemic stroke (AIS) is one of the leading causes of death and disability worldwide, and a substantial burden is imposed on healthcare systems (1, 2). Post-stroke epilepsy (PSE) is observed in approximately 2%–4% of AIS survivors and is associated with higher rates of rehospitalization and poorer functional outcomes (3–5). Therefore, modifiable risk factors should be identified to develop prevention strategies and to guide clinical decision-making.

Atherosclerosis underlies the pathogenesis of the majority of AIS cases (6). Dyslipidemia not only promotes atherosclerosis but also modulates systemic metabolism and inflammatory responses (7). Perturbations in lipid metabolism have been implicated in several pathophysiological processes relevant to epilepsy. These processes include ferroptosis, lipid-droplet/autophagy-related pathways (lipophagy), and gut microbiota–mediated immune regulation (8). Guo et al. (9) reported that pediatric patients with epilepsy had markedly lower serum high-density lipoprotein cholesterol (HDL) concentrations than healthy children and were at higher risk of dyslipidemia. By contrast, Assis et al. (10) observed an inverse association between dyslipidemia and status epilepticus in elderly patients with epilepsy. Because these studies examined different age groups and clinical outcomes, the association between dyslipidemia and PSE among middle-aged and elderly patients with AIS remains unclear.

The atherogenic index of plasma (AIP) is a simple metric used to reflect dysregulated lipid metabolism. AIP integrates triglycerides (TG) and HDL. This integration captures the balance between pro-atherosclerotic (TG-rich lipoprotein) and anti-atherosclerotic (HDL) components. The overall disordered lipid metabolic status is better characterized by AIP than by individual lipid indicators. Prediction of adverse cardiovascular and stroke outcomes has been shown for AIP (11, 12). Elevated AIP has been associated with unfavorable post-stroke outcomes, including depression, early neurological deterioration, poor functional recovery, and increased mortality (13–16). The association between AIP and PSE among middle-aged and elderly patients with AIS remains unclear. The association between AIP and PSE, including potential mediating pathways and interaction effects, should be elucidated to inform prevention strategies and risk stratification. The study population was defined as people aged 45 years and older because lipid abnormalities are more pronounced and stroke is the main cause of acquired epilepsy in this group (17).

This study had three primary aims. First, to evaluate the association between AIP and PSE occurring within one year after AIS, including assessment of dose–response and potential threshold effects, and to explore sex-specific differences. Second, to assess the mediating role of fibrinogen in the relationship between AIP and PSE. Third, to investigate whether additive or multiplicative interaction effects exist across relevant subgroups.

## 2 Methods

### 2.1 Data sources

The data used in this study are available in the Dryad Digital Repository^1^ (18). The original authors processed the missing data prior to deposition. Dryad provides open access to the original dataset. The original data collection followed ethical standards. The reuse of the data followed open-data guidelines.

### 2.2 Study design and participants

The data for this study were obtained from a database established by the Chongqing Emergency Medical Center. Between June 2017 and June 2022, the investigators conducted a retrospective observational study to extract records for 42,079 patients with stroke. After applying rigorous inclusion and exclusion criteria, 21,459 patients with AIS remained for analysis and for development of a predictive model for PSE. PostgreSQL was used as the database management system and multidimensional clinical data were integrated via Structured Query Language (SQL).

Inclusion criteria required a clinical diagnosis of acute ischemic stroke and hospitalization for treatment. Exclusion criteria were as follows: (1) prior stroke or transient ischemic attack; (2) prior traumatic brain injury, intracranial tumor, or cerebrovascular malformation that could cause seizures; (3) preexisting epilepsy or prior use of antiepileptic drugs for seizure prophylaxis or other indications such as migraine or psychiatric disorders; (4) death within 72 h of stroke onset; and (5) loss to follow-up, defined as no outpatient records or inability to contact by telephone, or death within 3 months of the index stroke.

**Figure 1:**
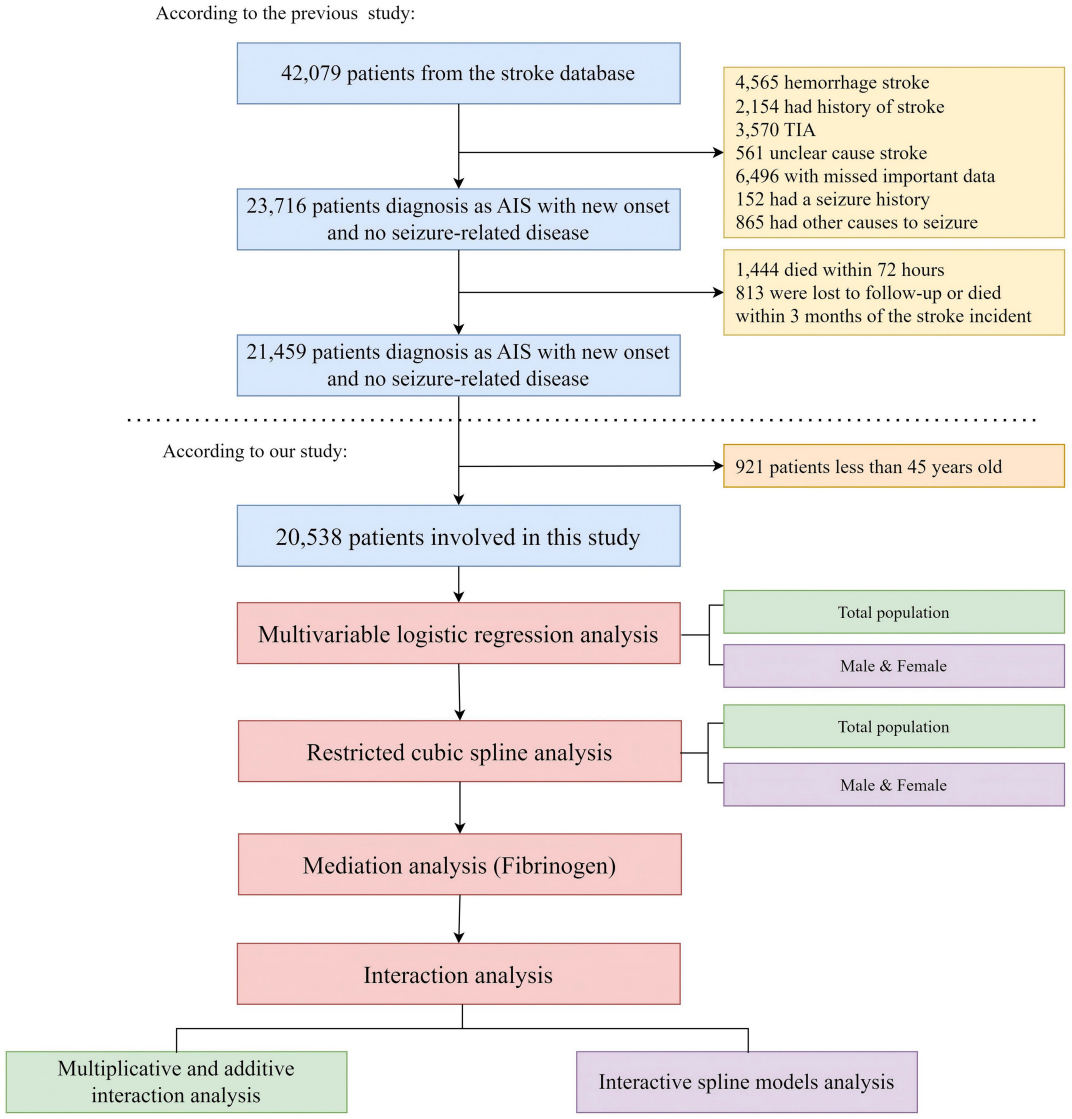
The overall flowchart of the study.

For the current analysis, the cohort was further limited to patients aged 45 to 90 years to focus on middle-aged and older adults, a group in which dyslipidemia and stroke-related acquired epilepsy are more common. As shown in Figure 1, application of the selection criteria resulted in a final cohort of 20,538 patients.

The study protocol received approval from the Ethics Committee of Chongqing University Center Hospital. Informed consent was waived because the dataset was anonymized and the study complied with applicable regulatory standards.

### 2.3 Variables

Potential covariates were selected based on clinical relevance and prior studies (18–31), including age, sex, National Institutes of Health Stroke Scale (NIHSS), cerebral herniation, hydrocephalus, deep vein thrombosis, diabetes, hypertension, coronary disease, atrial fibrillation, fatty liver, cortical involvement, large vessel disease, platelet count, white blood cell count, hemoglobin A1c (HbA1c), C-reactive protein (CRP), alanine aminotransferase, bilirubin, albumin, urea, estimated glomerular filtration rate (eGFR), and blood uric acid. Demographic and clinical variables were extracted from the hospital database. Imaging findings were taken from CT and MRI reports. Cortical involvement was recorded when CT or MRI showed infarction in the frontal, parietal, temporal, occipital, or insular lobe. Vascular imaging (CTA, MRA, or DSA) was reviewed to identify large vessel disease. Laboratory indicators were extracted from hospital records. These included TG, HDL, platelet count, white blood cell count, HbA1c, CRP, alanine aminotransferase, bilirubin, albumin, urea, creatinine, and blood uric acid. The eGFR was calculated using the modified Modification of Diet in Renal Disease equation for Chinese patients (32).

The primary outcome was PSE within one year after AIS. PSE was identified during follow-up and confirmed through outpatient re-evaluations and telephone interviews conducted by neurologists. TG and HDL were measured in the hospital laboratory. The AIP was calculated as follows (33). Participants were split at the median AIP (0.079) into two groups. Group Q1 had AIP < 0.079. Group Q2 had AIP ≥ 0.079. Splitting at the median preserves group balance and supports comparison of risk between lower and higher AIP.


$$\mathrm{AIP}=\log_{10}\;(\frac{\mathrm{TG}}{\mathrm{HDL}})$$

### 2.4 Statistical analysis

Normality of the variables was assessed using histogram distributions and mean-to-standard deviation (SD) ratio (34). All nearly normally distributed continuous variables were expressed as mean ± SD. Highly skewed continuous variables were represented as median with interquartile range (IQR). Only CRP exhibited highly skewed continuous (Supplementary Figure S1, Table S1). Categorical variables were reported as frequencies and percentages (%). Comparisons of continuous variables between groups were performed using the independent samples Student’s *t*-test or the Mann–Whitney *U*-test, depending on distribution normality. Categorical data were compared using the chi-square test as appropriate.

### 2.5 Association between AIP and PSE

The primary association between AIP and PSE was tested by multivariable logistic regression. Results are reported as adjusted odds ratios (OR) with 95% confidence intervals (CI). AIP was studied both as a standardized continuous variable (per 1-SD increase) and as a categorical variable (Q1 reference). Model 1 was adjusted for age, sex, and NIHSS. Model 2 was adjusted for age, sex, NIHSS, cerebral herniation, hydrocephalus, deep vein thrombosis, diabetes, hypertension, coronary disease, atrial fibrillation, and fatty liver. Model 3 was adjusted for age, sex, NIHSS, cerebral herniation, hydrocephalus, deep vein thrombosis, diabetes, hypertension, coronary disease, atrial fibrillation, fatty liver, cortical involvement, large vessel disease, platelet count, white blood cell count, HbA1c, CRP, alanine aminotransferase, bilirubin, albumin, urea, eGFR, and blood uric acid. Variance inflation factors were checked and found to be <5 for all covariates, indicating no problematic multicollinearity. Restricted cubic spline (RCS) with four knots (5th, 35th, 65th, 95th percentiles) was used to assess dose–response relations and nonlinearity. Two-piecewise logistic regression with smoothing was used to find potential threshold effects. The likelihood ratio test and bootstrap resampling were used for validation of nonlinear and threshold findings.

### 2.6 Mediation, interaction and sensitivity analyses

Mediation analysis methods included the Sobel test, bootstrap mediation, and the quasi-Bayesian Monte Carlo method with 1,000 simulations. Interaction analyses were done to test effect modification by key covariates: age (<65 vs. ≥ 65 years), sex, NIHSS (<12 vs. ≥ 12), cortical involvement, CRP (≤3 mg/L vs. > 3 mg/L), and HbA1c (<6.5% vs. ≥ 6.5%). Multiplicative interaction was tested by likelihood ratio test. Additive interaction was assessed with relative excess risk due to interaction (RERI) and attributable proportion (AP). For continuous modifiers (HbA1c, CRP, NIHSS), interactive spline models were fitted using 3 knots (10th, 50th, 90th percentiles) through the R package interaction RCS (R Project for Statistical Computing). Sensitivity analyses included models using AIP per 0.1 unit and AIP tertiles. Robustness to unmeasured confounding was assessed by calculating *E*-values.

All analyses were conducted using R Statistical Software (Version 4.2.2, http://www.R-project.org, The R Foundation) and Free Statistics Analysis Platform (Version 2.3beta, Beijing, China) (35). A two-tailed *p*-value < 0.05 was considered statistically significant.

## 3 Results

### 3.1 Baseline characteristics of the study population

Table 1 shows the baseline characteristics of the 20,538 study participants by AIP group. The mean age was 67.69 ± 10.93 years. The proportion of males was 50.04%. Compared with the lower AIP group (Q1), individuals in the higher AIP group (Q2) were younger and more often female. They were more likely to have diabetes, hypertension, coronary disease, and fatty liver. They were less likely to have atrial fibrillation, cerebral herniation, hydrocephalus, and deep vein thrombosis than those in Q1. In addition, they had higher platelet count, HbA1c, alanine aminotransferase, albumin, urea, and blood uric acid. They had lower NIHSS, white blood cell count, CRP, and bilirubin than those in Q1.

**Table 1:** Characteristics of study population.

Variables	Total (*n* = 20,538)	AIP		*p*
		Q1 (<0.079, *n* = 10,261)	Q2 (≥0.079, *n* = 10,277)	
Age (years), mean ± SD	67.69 ± 10.93	69.90 ± 11.21	65.49 ± 10.18	<0.001
Sex, *n* (%)				0.029
Male	10,278 (50.04)	5,213 (50.80)	5,065 (49.28)	
Female	10,260 (49.96)	5,048 (49.20)	5,212 (50.72)	
NIHSS, mean ± SD	8.05 ± 2.96	8.97 ± 3.02	7.14 ± 2.59	<0.001
Cerebral herniation, *n* (%)	165 (0.80)	101 (0.98)	64 (0.62)	0.004
Hydrocephalus, *n* (%)	250 (1.22)	173 (1.69)	77 (0.75)	<0.001
Deep vein thrombosis, *n* (%)	1,296 (6.31)	841 (8.20)	455 (4.43)	<0.001
Diabetes, *n* (%)	7,167 (34.90)	1,924 (18.75)	5,243 (51.02)	<0.001
Hypertension, *n* (%)	14,308 (69.67)	5,980 (58.28)	8,328 (81.04)	<0.001
Coronary disease, *n* (%)	9,521 (46.36)	4,583 (44.66)	4,938 (48.05)	<0.001
Atrial fibrillation, *n* (%)	2,024 (9.85)	1,322 (12.88)	702 (6.83)	<0.001
Fatty liver, *n* (%)	4,077 (19.85)	833 (8.12)	3,244 (31.57)	<0.001
Cortical involvement, *n* (%)	1,255 (6.11)	702 (6.84)	553 (5.38)	<0.001
Large vessel disease, *n* (%)	5,401 (26.30)	2,505 (24.41)	2,896 (28.18)	<0.001
Platelet count (10^9^/L), mean ± SD	189.62 ± 26.86	186.71 ± 24.50	192.52 ± 28.74	<0.001
White blood cell count (10^9^/L), mean ± SD	8.40 ± 1.49	8.49 ± 1.50	8.30 ± 1.48	<0.001
HbA1c (%), mean ± SD	6.69 ± 0.93	6.42 ± 0.75	6.96 ± 1.01	<0.001
CRP (mg/L), median (IQR)	9.50 (4.80, 19.30)	10.90 (4.80, 24.30)	8.50 (4.70, 16.70)	<0.001
Alanine aminotransferase (U/L), mean ± SD	24.28 ± 10.29	23.55 ± 9.08	25.01 ± 11.33	<0.001
Bilirubin (μmol/L), mean ± SD	15.22 ± 4.02	16.59 ± 4.30	13.85 ± 3.19	<0.001
Albumin (g/L), mean ± SD	40.84 ± 2.25	40.53 ± 2.32	41.15 ± 2.13	<0.001
Urea (mmol/L), mean ± SD	6.47 ± 1.41	6.34 ± 1.12	6.61 ± 1.64	<0.001
eGFR (mL/min/1.73 m^2^), mean ± SD	88.82 ± 29.20	90.20 ± 28.16	87.44 ± 30.15	<0.001
Blood uric acid (μmol/L), mean ± SD	343.15 ± 58.62	327.36 ± 49.58	358.92 ± 62.57	<0.001
Fibrinogen (g/L), mean ± SD	3.61 ± 0.47	3.63 ± 0.47	3.59 ± 0.46	<0.001
Outcome				
PSE, *n* (%)	867 (4.22)	300 (2.92)	567 (5.52)	<0.001

AIP, atherogenic index of plasma; PSE, post-stroke epilepsy; NIHSS, National Institutes of Health Stroke Scale; HbA1c, hemoglobin A1c; CRP, C-reactive protein; eGFR, estimated glomerular filtration rate.

### 3.2 Association between AIP and PSE in the total population

Table 2 shows that AIP was significantly and positively associated with PSE risk. In the fully adjusted model (Model 3), in continuous analysis, each 1-SD increase in AIP was associated with a 183% higher risk of PSE (OR = 2.83; 95% CI: 2.53–3.18; *p* < 0.001). In the two-group analysis, compared with Q1, Q2 had a 340% higher risk of PSE (OR = 4.40; 95% CI: 3.46–5.59; *p* < 0.001).

**Table 2:** Association between AIP and PSE in different models.

Variable	Crude		Model 1		Model 2		Model 3	
	OR (95% CI)	*p*	OR (95% CI)	*p*	OR (95% CI)	*p*	OR (95% CI)	*p*
Total population								
AIP per 1-SD	1.27 (1.19 ~ 1.36)	<0.001	2 (1.85 ~ 2.16)	<0.001	2 (1.84 ~ 2.17)	<0.001	2.83 (2.53 ~ 3.18)	<0.001
AIP (Q1 < 0.079)	1 (Ref)		1 (Ref)		1 (Ref)		1 (Ref)	
AIP (Q2 ≥ 0.079)	1.94 (1.68 ~ 2.24)	<0.001	4.02 (3.40 ~ 4.76)	<0.001	3.85 (3.22 ~ 4.60)	<0.001	4.40 (3.46 ~ 5.59)	<0.001
Males								
AIP per 1-SD	1.31 (1.18 ~ 1.45)	<0.001	1.72 (1.55 ~ 1.92)	<0.001	1.64 (1.45 ~ 1.85)	<0.001	2.64 (2.17 ~ 3.2)	<0.001
AIP (Q1 < 0.067)	1 (Ref)		1 (Ref)		1 (Ref)		1 (Ref)	
AIP (Q2 ≥ 0.067)	2.15 (1.70 ~ 2.72)	<0.001	4.25 (3.24 ~ 5.56)	<0.001	3.91 (2.92 ~ 5.24)	<0.001	7.99 (5.10 ~ 12.51)	<0.001
Females								
AIP per 1-SD	1.25 (1.14 ~ 1.36)	<0.001	2.68 (2.38 ~ 3.02)	<0.001	2.98 (2.6 ~ 3.4)	<0.001	3.17 (2.68 ~ 3.75)	<0.001
AIP (Q1 < 0.084)	1 (Ref)		1 (Ref)		1 (Ref)		1 (Ref)	
AIP (Q2 ≥ 0.084)	1.84 (1.53 ~ 2.21)	<0.001	4.94 (3.92 ~ 6.21)	<0.001	5.18 (4.05 ~ 6.62)	<0.001	4.35 (3.16 ~ 6.00)	<0.001

Model 1: Adjusted for age, sex (for total population only), and NIHSS.

Model 2: Adjusted for age, sex (for total population only), NIHSS, cerebral herniation, hydrocephalus, deep vein thrombosis, diabetes, hypertension, coronary disease, atrial fibrillation, and fatty liver.

Model 3: Adjusted for age, sex (for total population only), NIHSS, cerebral herniation, hydrocephalus, deep vein thrombosis, diabetes, hypertension, coronary disease, atrial fibrillation, fatty liver, cortical involvement, large vessel disease, platelet count, white blood cell count, HbA1c, CRP, alanine aminotransferase, bilirubin, albumin, urea, eGFR, and blood uric acid. OR, odds ratio; CI, confidence interval; SD, standard deviation; AIP, atherogenic index of plasma; PSE, post-stroke epilepsy; NIHSS, National Institutes of Health Stroke Scale; HbA1c, Hemoglobin A1c; CRP, C-reactive protein; eGFR, estimated glomerular filtration rate.

**Figure 2:**
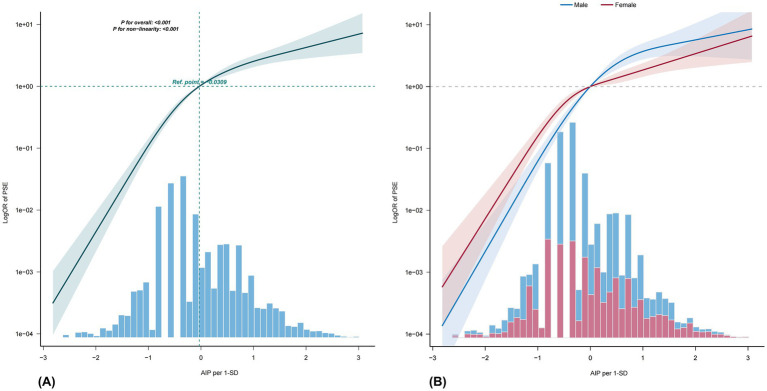
Restricted cubic spline curve in total population **(A)**. Adjusted for age, sex, NIHSS, cerebral herniation, hydrocephalus, deep vein thrombosis, diabetes, hypertension, coronary disease, atrial fibrillation, fatty liver, cortical involvement, large vessel disease, platelet count, white blood cell count, HbA1c, CRP, alanine aminotransferase, bilirubin, albumin, urea, eGFR, and blood uric acid. Restricted cubic spline curve in males and females **(B)**. Adjusted for age, NIHSS, cerebral herniation, hydrocephalus, deep vein thrombosis, diabetes, hypertension, coronary disease, atrial fibrillation, fatty liver, cortical involvement, large vessel disease, platelet count, white blood cell count, HbA1c, CRP, alanine aminotransferase, bilirubin, albumin, urea, eGFR, and blood uric acid. AIP, atherogenic index of plasma; PSE, post-stroke epilepsy; NIHSS, National Institutes of Health Stroke Scale; HbA1c, Hemoglobin A1c; CRP, C-reactive protein; eGFR, estimated glomerular filtration rate.

RCS curves for the total population are shown in Figure 2A. A test for nonlinearity indicated a significant nonlinear relationship between AIP and PSE, with a threshold at 0.048 (Table 3). When AIP < 0.048, each 1-SD increase in AIP was associated with a much higher odds of PSE (OR = 10.75; 95% CI: 6.24–18.52; *p* < 0.001). When AIP ≥ 0.048, the association remained significant but was weaker (OR = 2.22; 95% CI: 1.77–2.79; *p* < 0.001).

**Table 3:** Threshold effect analysis of the association between AIP and PSE.

Subgroup	AIP	*n*	OR (95% CI) per 1-SD	*p*	Likelihood ratio test
Total population	<0.048	8,296	10.75 (6.24 ~ 18.52)	<0.001	<0.001
	≥0.048	12,053	2.22 (1.77 ~ 2.79)	<0.001	
Males	<0.049	4,146	19.08 (6.78 ~ 53.69)	<0.001	<0.001
	≥0.049	6,029	2.59 (1.66 ~ 4.05)	<0.001	
Females	<0.026	3,285	9.67 (3.66 ~ 25.57)	<0.001	<0.001
	≥0.026	6,886	2.34 (1.77 ~ 3.11)	<0.001	

AIP was truncated at the 0.5% ~ 99.5% percentiles to exclude extreme values. Adjusted for age, sex (for total population only), NIHSS, cerebral herniation, hydrocephalus, deep vein thrombosis, diabetes, hypertension, coronary disease, atrial fibrillation, fatty liver, cortical involvement, large vessel disease, platelet count, white blood cell count, HbA1c, CRP, alanine aminotransferase, bilirubin, albumin, urea, eGFR, and blood uric acid.

OR, odds ratio; CI, confidence interval; SD, standard deviation; AIP, atherogenic index of plasma; PSE, post-stroke epilepsy; NIHSS, National Institutes of Health Stroke Scale; HbA1c, Hemoglobin A1c; CRP, C-reactive protein; eGFR, estimated glomerular filtration rate.

### 3.3 Association between AIP and PSE in the sex-stratified population

The 2D heatmap (Supplementary Figure S2) shows that the relationship between AIP and PSE in both male and female increases first and then slightly decreases with rising AIP. The increase was faster and the decrease was slower in male. The sex-stratified analysis in Table 2 indicates that high AIP is associated with an increased risk of PSE. After adjusting for covariates (Model 3), the PSE risk in male with high AIP was 7.99 times that of the low AIP group (95% CI: 5.10–12.51), and 4.35 times higher in female (95% CI: 3.16–6.00). The RCS curve in Figure 2B and Table 3 show that both male and female exhibit significant threshold effects. The inflection point for male was 0.049; below this value, the adjusted OR for each 1-SD increase in AIP was 19.08 (95% CI: 6.78–53.69; *p* < 0.001), and 2.59 (95% CI: 1.66–4.05; *p* < 0.001) when equal to or above this value. For female, the inflection point was 0.026; below this value, the OR was 9.67 (95% CI: 3.66–25.57; *p* < 0.001), and 2.34 (95% CI: 1.77–3.11; *p* < 0.001) when equal to or above this value.

### 3.4 Mediation analysis

**Figure 3:**
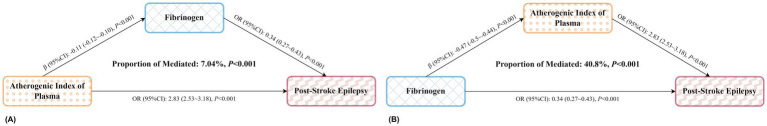
Mutual mediation of PSE by fibrinogen and AIP. The association between AIP and PSE was partially mediated by fibrinogen **(A)**. The association between fibrinogen and PSE was partially mediated by AIP **(B)**. Adjusted for age, sex, NIHSS, cerebral herniation, hydrocephalus, deep vein thrombosis, diabetes, hypertension, coronary disease, atrial fibrillation, fatty liver, cortical involvement, large vessel disease, platelet count, white blood cell count, HbA1c, CRP, alanine aminotransferase, bilirubin, albumin, urea, eGFR, and blood uric acid. OR, odds ratio; CI, confidence interval; SD, standard deviation; AIP, atherogenic index of plasma; PSE, post-stroke epilepsy; NIHSS, National Institutes of Health Stroke Scale; HbA1c, Hemoglobin A1c; CRP, C-reactive protein; eGFR, estimated glomerular filtration rate.

Figure 3 shows the potential mediating role of fibrinogen in the association between AIP and PSE. We found that a higher AIP was associated with lower fibrinogen levels (*β* = −0.11, 95% CI: −0.12 to −0.10). Meanwhile, higher fibrinogen levels were significantly associated with lower risk of PSE (OR = 0.34, 95% CI: 0.27 to 0.43). Mediation analysis (Figure 3A) indicated that the association between AIP and PSE was partially mediated by fibrinogen, with a mediation proportion of 7.04% (*p* < 0.001). Figure 3B showed that AIP mediated 40.8% of the effect of fibrinogen on PSE (*p* < 0.001). This mutual mediation suggests a synergistic effect between AIP and fibrinogen, indicating that their combined impact on PSE may be more significant than their individual effects.

### 3.5 Interaction analysis

**Figure 4:**
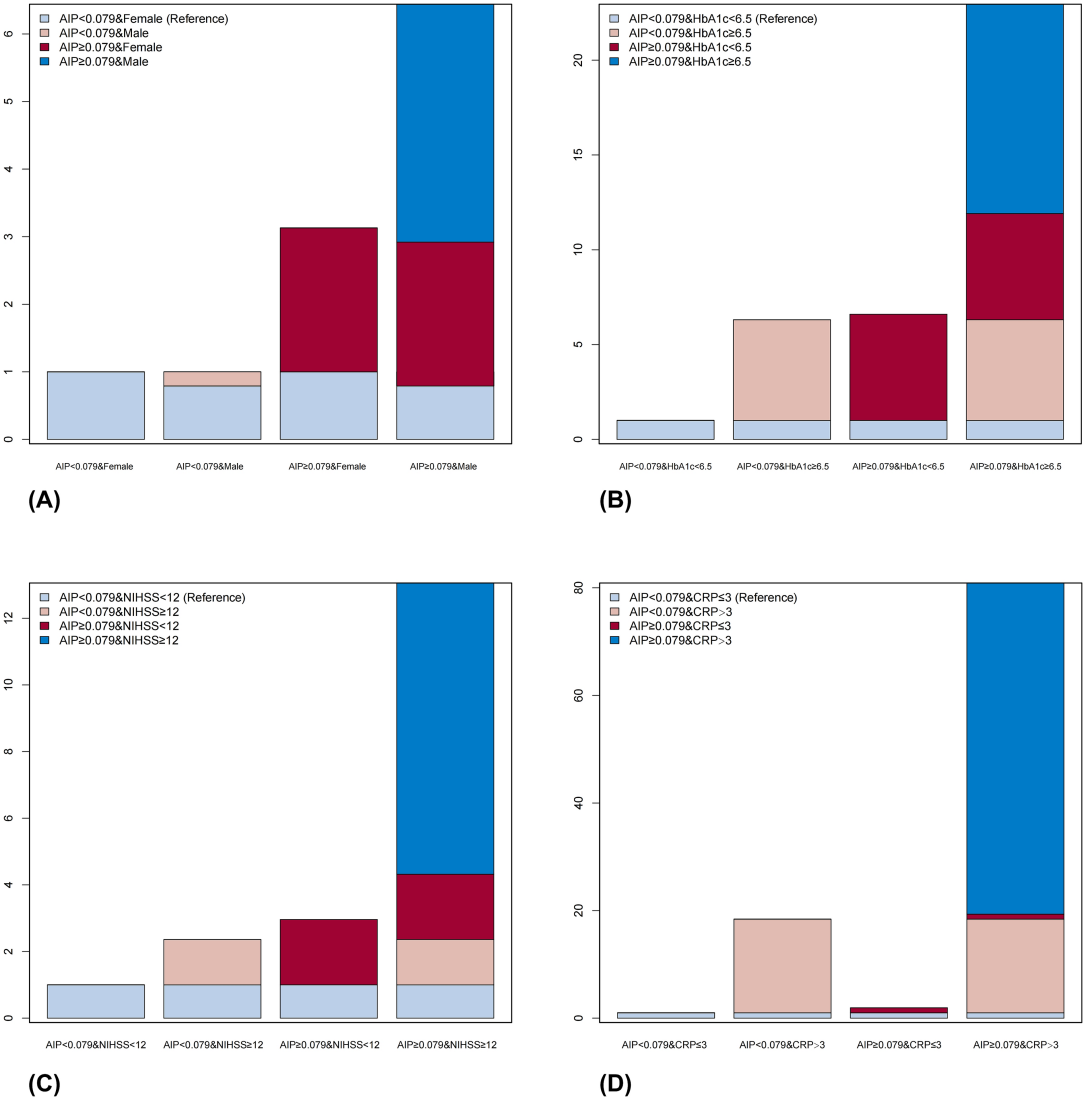
Additive interactions between sex, HbA1c, NIHSS, CRP, and AIP on PSE. Adjusted for age, NIHSS, cerebral herniation, hydrocephalus, deep vein thrombosis, diabetes, hypertension, coronary disease, atrial fibrillation, fatty liver, cortical involvement, large vessel disease, platelet count, white blood cell count, HbA1c, CRP, alanine aminotransferase, bilirubin, albumin, urea, eGFR, and blood uric acid **(A)**. Adjusted for age, sex, NIHSS, cerebral herniation, hydrocephalus, deep vein thrombosis, diabetes, hypertension, coronary disease, atrial fibrillation, fatty liver, cortical involvement, large vessel disease, platelet count, white blood cell count, CRP, alanine aminotransferase, bilirubin, albumin, urea, eGFR, and blood uric acid **(B)**. Adjusted for age, sex, cerebral herniation, hydrocephalus, deep vein thrombosis, diabetes, hypertension, coronary disease, atrial fibrillation, fatty liver, cortical involvement, large vessel disease, platelet count, white blood cell count, HbA1c, CRP, alanine aminotransferase, bilirubin, albumin, urea, eGFR, and blood uric acid **(C)**. Adjusted for age, sex, NIHSS, cerebral herniation, hydrocephalus, deep vein thrombosis, diabetes, hypertension, coronary disease, atrial fibrillation, fatty liver, cortical involvement, large vessel disease, platelet count, white blood cell count, HbA1c, alanine aminotransferase, bilirubin, albumin, urea, eGFR, and blood uric acid **(D)**. AIP, atherogenic index of plasma; PSE, post-stroke epilepsy; NIHSS, National Institutes of Health Stroke Scale; HbA1c, Hemoglobin A1c; CRP, C-reactive protein; eGFR, estimated glomerular filtration rate.

**Figure 5:**
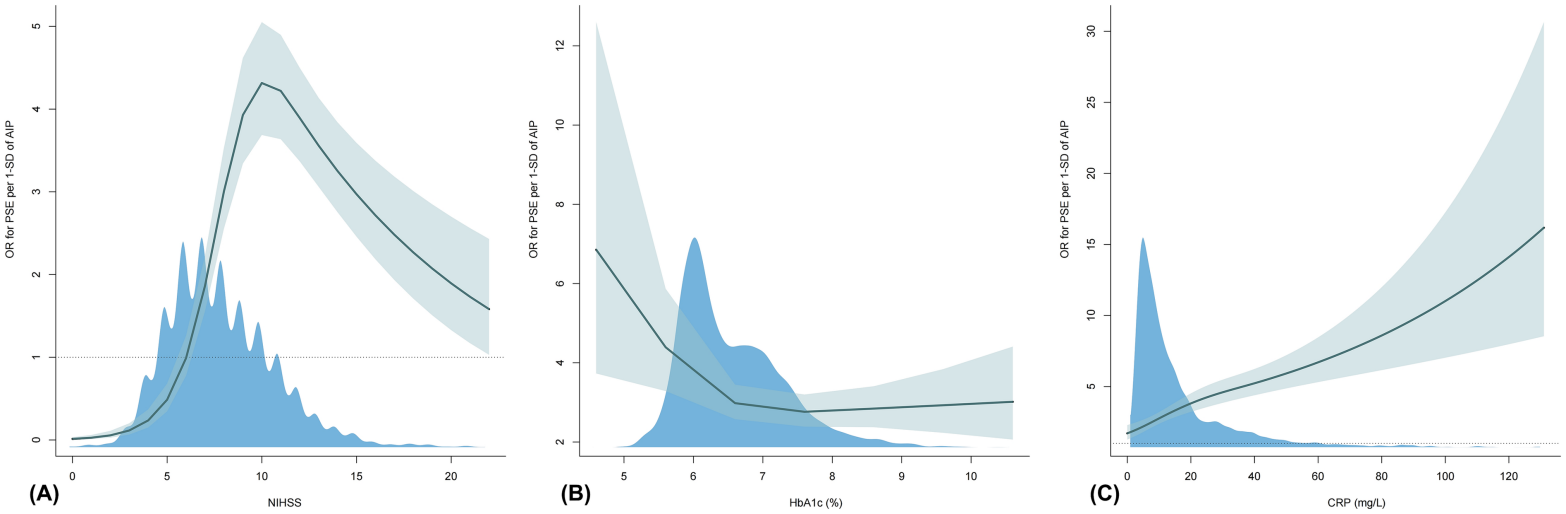
OR for PSE by per 1-SD of AIP as a function of NIHSS, HbA1c and CRP. Adjusted for age, sex, NIHSS, cerebral herniation, hydrocephalus, deep vein thrombosis, diabetes, hypertension, coronary disease, atrial fibrillation, fatty liver, cortical involvement, large vessel disease, platelet count, white blood cell count, CRP, alanine aminotransferase, bilirubin, albumin, urea, eGFR, blood uric acid **(A)**. Adjusted for age, sex, NIHSS, cerebral herniation, hydrocephalus, deep vein thrombosis, diabetes, hypertension, coronary disease, atrial fibrillation, fatty liver, cortical involvement, large vessel disease, platelet count, white blood cell count, HbA1c, alanine aminotransferase, bilirubin, albumin, urea, eGFR, and blood uric acid **(B)**. Adjusted for age, sex, NIHSS, cerebral herniation, hydrocephalus, deep vein thrombosis, diabetes, hypertension, coronary disease, atrial fibrillation, fatty liver, cortical involvement, large vessel disease, platelet count, white blood cell count, HbA1c, CRP, alanine aminotransferase, bilirubin, albumin, urea, eGFR, and blood uric acid **(C)**. OR, odds ratio; SD, standard deviation; AIP, atherogenic index of plasma; PSE, post-stroke epilepsy; NIHSS, National Institutes of Health Stroke Scale; HbA1c, Hemoglobin A1c; CRP, C-reactive protein; eGFR, estimated glomerular filtration rate.

Table 4 and Figure 4 show the combined effects of AIP with sex, HbA1c, NIHSS, and CRP on PSE. Compared to reference group (female; AIP < 0.079), OR for PSE risk in male with AIP ≥ 0.079 was 6.43 (95% CI: 4.58–9.03; *p* < 0.001). When AIP ≥ 0.079 and HbA1c ≥ 6.5%, the OR reached 22.96 (95% CI: 15.58–33.82). For AIP ≥ 0.079 and NIHSS ≥ 12, the OR was 13.06 (95% CI: 9.04–18.88). When CRP > 3 mg/L and AIP ≥ 0.079, the OR increased to 80.91 (95% CI: 30.05–217.83; *p* < 0.05). AIP exhibited significant multiplicative and additive interaction effects with the first three factors (sex, HbA1c, NIHSS), with RERI of 3.52 (95% CI: 1.75–5.28), 11.04 (95% CI: 4.94–17.15), and 8.74 (95% CI: 4.36–13.12), respectively. A significant additive interaction was found only with CRP, with RERI of 61.56 (95% CI: 0.54–122.58). The interaction spline plots showed that AIP was associated with PSE across all ranges of each indicator. The association was stronger at low HbA1c, moderate NIHSS, and high CRP levels (Figure 5). The relationship between AIP and PSE was consistent across age groups and by cortical involvement (Supplementary Table S2).

**Table 4:** Multiplicative and additive interactions between AIP and clinical characteristics on the risk of PSE.

Subgroup		AIP	OR (95% CI)	Multiplicative interaction	Additive interaction	
				*P*	RERI (95% CI)	AP (95% CI)
Sex	Female	Q1	1 (Ref)	<0.001	3.52 (1.75 ~ 5.28)	0.55 (0.41 ~ 0.68)
		Q2	3.13 (2.37 ~ 4.12)*			
	Male	Q1	0.79 (0.54 ~ 1.14)			
		Q2	6.43 (4.58 ~ 9.03)*			
NIHSS	<12	Q1	1 (Ref)	0.010	8.74 (4.36 ~ 13.12)	0.67 (0.56 ~ 0.78)
		Q2	2.96 (2.28 ~ 3.85)*			
	≥12	Q1	2.36 (1.73 ~ 3.21)*			
		Q2	13.06 (9.04 ~ 18.88)*			
CRP (mg/L)	≤3	Q1	1 (Ref)	0.400	61.56 (0.54 ~ 122.58)	0.76 (0.69 ~ 0.83)
		Q2	1.93 (0.29 ~ 13.03)			
	>3	Q1	18.41 (6.88 ~ 49.25)*			
		Q2	80.91 (30.05 ~ 217.83)*			
HbA1c (%)	≤6.5	Q1	1(Ref)	0.010	11.04 (4.94 ~ 17.15)	0.48 (0.35 ~ 0.61)
		Q2	6.60 (4.37 ~ 9.97)*			
	>6.5	Q1	6.31 (4.35 ~ 9.16)*			
		Q2	22.96 (15.58 ~ 33.82)*			

Adjusted for age, sex, NIHSS, cerebral herniation, hydrocephalus, deep vein thrombosis, diabetes, hypertension, coronary disease, atrial fibrillation, fatty liver, cortical involvement, large vessel disease, platelet count, white blood cell count, HbA1c, CRP, alanine aminotransferase, bilirubin, albumin, urea, eGFR, and blood uric acid. For subgroup analyses conducted within levels of a categorical variable, that variable was not included as a covariate in the subgroup-specific models. *p < 0.001. If the 95% CIs for RERI and AP do not include 0, the interaction between them is considered statistically significant. OR, odds ratio; CI, confidence interval; RERI, relative excess risk due to interaction; AP, attributable proportion; AIP, atherogenic index of plasma; PSE, post-stroke epilepsy; NIHSS, National Institutes of Health Stroke Scale; HbA1c, Hemoglobin A1c; CRP, C-reactive protein; eGFR, estimated glomerular filtration rate.

### 3.6 Sensitivity analysis

Sensitivity analysis results were stable (Supplementary Table S3). For the current study, the E-value is 5.11, and for the lower confidence interval limit it is 4.50. Therefore, the observed OR of 2.83 could be explained by an unmeasured confounding factor that is associated with both AIP and new PSE with an OR of 5.11 (beyond the measured confounders), whereas a weaker confounding factor could not explain it.

## 4 Discussion

Based on a retrospective analysis of 20,538 middle-aged and elderly patients with AIS, this study identified an association between AIP at admission and the risk of PSE within one year. The core results showed that elevated AIP was significantly positively correlated with PSE risk. After full adjustment for covariates, each 1-SD increase in AIP was associated with an 183% higher risk of PSE. The high AIP group (Q2 ≥ 0.079) had a 340% higher risk compared to the control group. A non-linear threshold effect was identified, with an overall inflection point at 0.048 (males: 0.049; females: 0.026), and the association between AIP and PSE was stronger below this threshold. Fibrinogen and AIP exerted bidirectional mediating effects in the occurrence of PSE. Additionally, AIP had significant multiplicative and additive interaction effects with sex, NIHSS, and HbA1c, and a significant additive interaction effect with CRP.

Deng et al. (13) focused on early-onset post-stroke depression within 2 weeks after AIS. Their study included 667 patients and confirmed that high AIP is an independent risk factor for early-onset post-stroke depression. Compared with the present study, both validated the prognostic value of AIP in stroke. However, there is a key difference in outcome types (early-onset depression vs. secondary epilepsy one year after stroke). This difference suggests that AIP may be associated with different types of post-stroke complications, such as neuropsychiatric and neurological outcomes. Tatar et al. (16) studied Turkish patients with AIS. Their endpoint was 1-month mortality. The core finding was that low AIP was an independent predictor of short-term mortality. The present study, in contrast, focused on PSE within one year after AIS. Thus, the two studies differ both in endpoint type and in the direction of the association between AIP and outcome. Liu et al. (15) used the endpoint of adverse functional outcomes (mRS 3–6) at 3 months after AIS. They confirmed that high AIP (threshold 0.112) was an independent predictor. Both that study and the present study highlight the prognostic value of AIP in AIS. But the outcome types are different. Liu et al. focused on functional impairment. The present study focused on neurological complications. The sample size of Liu et al. was 1,463, much smaller than the present study’s 20,538. No non-linear relationship between AIP and outcome was found in Liu et al. In contrast, the present study, using restricted cubic spline analysis, revealed a threshold effect of AIP on post-stroke epilepsy (inflection point 0.636 in the total population). The risk increase was more significant below the inflection point. In addition, the previous studies did not conduct stratified and subgroup analyses. The present study provides more refined stratification evidence for clinical practice.

As a logarithmic calculation of TG/HDL, elevated AIP directly reflects dyslipidemia characterized by increased triglycerides and decreased HDL. This abnormality leads to an imbalance in cerebral lipid components. On the one hand, increased levels of neutral lipids such as TG promote the accumulation of lipid droplets in astrocytes, forming lipid-accumulated reactive astrocytes (LARA). LARA enhances neuronal excitability by upregulating adenosine 2A receptor and reducing glutamate reuptake, ultimately promoting epilepsy progression (36). On the other hand, decreased HDL levels reduce the reverse cholesterol transport from peripheral tissues to the liver, leading to abnormal elevation of lipids such as cholesterol esters in the brain (37). Increased cholesterol esters have been confirmed to be closely associated with cell membrane remodeling and synaptic dysfunction in temporal lobe epilepsy models, which may further increase epilepsy susceptibility (38).

The mutual mediation indicates a synergistic effect between AIP and fibrinogen. Fibrinogen promotes atherogenesis; elevated AIP may reflect fibrinogen consumption in atherosclerotic lesions or functional impairment during plaque development (39). After stroke-induced blood–brain barrier (BBB) disruption, plasma fibrinogen leaks into the central nervous system (CNS), converts to fibrin, activates microglia, induces proinflammatory cytokine release, triggers inflammation, and disturbs the neural microenvironment (40). Fibrinogen also induces inhibitory long-term depression via β3 integrin activation to suppress inhibitory synaptic transmission; low fibrinogen levels attenuate this activation, leading to neuronal hyperexcitability and seizures (41). Atherosclerosis-driven vascular injury exacerbates fibrinogen-mediated neurotoxicity, and dysfunctional fibrinogen further accelerates atherogenesis, jointly amplifying CNS damage and increasing epilepsy risk.

The stronger correlation between elevated AIP and PSE risk in males is attributed to the absence of estrogen-mediated BBB protection. Estrogen (e.g., 17β-estradiol) reduces post-injury BBB permeability, alleviates ischemia-induced leakage, and inhibits the invasion of toxic and inflammatory factors into brain tissue (42). It also enhances PI3K regulatory subunit binding to IRS-1, activates the PI3K pathway, and mitigates ischemia, inflammation, and oxidative stress (43). In females, the synergistic neuroprotection of estrogen and IGF-1 activates the PI3K/Akt pathway, which promotes neuronal survival, inhibits GSK3β phosphorylation and apoptosis, and alleviates post-stroke neuronal degeneration and epilepsy-related inflammation (42).

NIHSS reflects stroke severity; high scores correlate with large infarct volume and cortical involvement, key drivers of PSE (44). Coexisting elevated AIP and high NIHSS exert additive pathophysiological effects: AIP-related lipid metabolic derangement, oxidative stress, and inflammation amplify neuronal susceptibility to abnormal discharge induced by severe brain injury, sharply increasing PSE risk. Moderate NIHSS corresponds to intermediate brain damage with an ischemic penumbra; timely reperfusion can limit infarct expansion (45). In such cases, parenchymal injury alone does not determine PSE risk, but AIP-associated lipid disturbances readily induce synaptic hyperexcitability, disrupt plasticity, and exacerbate penumbral metabolic dysfunction, synergizing with structural injury to elevate PSE risk (46). The positive additive interaction between AIP and HbA1c may arise from their synergistic amplification effects in energy metabolic disorders and neuroinflammatory pathways. Chronic hyperglycemia induces excessive activation of microglia, and the pro-inflammatory factors released by microglia further disrupt the integrity of the BBB and exacerbate synaptic dysfunction (47). The interaction curve shows a stronger association between AIP and PSE at low HbA1c levels, which may be because, under low HbA1c conditions, brain tissue energy metabolism is relatively dependent on glucose supply, and AIP elevation-induced lipid metabolic disorders more significantly interfere with the utilization of alternative energy substrates (such as ketone bodies and lactate), aggravating the imbalance between energy supply and demand (48). The additive interaction between AIP and CRP in predicting PSE risk stems from the convergence of inflammation and lipid metabolic dysregulation. Inflammation is closely linked to seizure occurrence (21). Systemic inflammation activates glial cells, releases proinflammatory cytokines and excitatory transmitters, and lowers the seizure threshold; as a core systemic inflammation marker, elevated CRP potentiates seizure risk via these pathways (49). Concurrent elevation of CRP and AIP leads to additive perturbations of the cerebral microenvironment through inflammation-driven neuronal injury and AIP-related lipid disorders, increasing neuronal hyperexcitability. Notably, this synergistic effect is positively correlated with CRP concentration, with higher CRP amplifying the interaction and markedly elevating PSE risk.

The findings support the use of the admission AIP as a screening marker for PSE, with risk stratification based on sex-specific cutoffs (male 0.049; female 0.026), and indicate that a wider array of clinical subgroup characteristics should be incorporated into risk assessment and intervention decisions. Overall, AIP exhibited a nonlinear threshold relationship with PSE (overall threshold 0.048), and the risk was markedly amplified when elevated AIP co-occurred with other high-risk factors. For example, AIP ≥ 0.079 combined with HbA1c ≥ 6.5% yielded OR of 22.96; AIP ≥ 0.079 with NIHSS ≥ 12 yielded OR = 13.06; and concurrent elevation of CRP (>3 mg/L) and AIP produced an even larger risk (OR = 80.91). Statistically, significant multiplicative and additive interactions were observed between AIP and sex, HbA1c, and NIHSS (RERI for sex, HbA1c, and NIHSS were 3.52, 11.04, and 8.74, respectively), whereas the interaction between AIP and CRP was predominantly additive (RERI = 61.56). These results suggest that more aggressive early-warning measures and targeted interventions are warranted for patients who are male, have severe neurological deficits, exhibit marked inflammatory response, or have poor glycemic control—examples include intensified lipid, glucose, and inflammation management, more frequent neurological surveillance, and EEG monitoring when indicated. By contrast, stratification by age and by presence or absence of cortical involvement did not materially alter the AIP–PSE association, implying that AIP retains prognostic value across age groups and cortical-involvement statuses. Clinically, the above subgroup-specific risks can inform individualized management plans; however, because this work is retrospective and observational, specific preventive and therapeutic strategies should be validated in prospective studies.

Our study had several important strengths. These included a large sample of 20,538 participants, a comprehensive assessment of the nonlinear dose–response and threshold effects of AIP on PSE, analysis of fibrinogen-mediated bidirectional mediation, and systematic exploration of multiplicative and additive interactions across multiple clinical subgroups. Despite these strengths, several limitations merit discussion and future attention. First, the retrospective design relied on existing medical records and secondary data. This limited us to variables that were recorded and may have introduced bias. Selection bias is one possibility. Patients could have been excluded because of early death, short-term loss to follow-up after discharge, or incomplete records. PSE ascertainment that relied on clinical records or follow-up calls may have introduced misclassification bias. AIP was calculated from a single lipid measurement taken at admission, so temporal changes in lipid profiles were not captured and measurement bias may have occurred. Some important confounders, notably medication use, were not available and could not be adjusted for. We calculated an E-value of 5.11 to gauge the potential impact of unmeasured confounding. This suggests some robustness of the associations, but residual confounding remains possible. Second, the cohort was recruited from Southwest China. Regional, ethnic, dietary, and healthcare differences may limit the generalizability. Replication in multiethnic and multicenter cohorts is necessary. Third, several subgroup interaction estimates were imprecise. For example, the RERI in the CRP subgroup ranged from 0.54 to 122.58. In subgroups where interaction effects by age stratification or cortical involvement were not significant, the 95% confidence intervals for RERI and AP were wide. Although interactions by sex, NIHSS, and HbA1c showed narrower intervals, their clinical relevance and external validity remain uncertain. These imprecisions likely reflect unequal subgroup sizes, baseline heterogeneity, and limited power for interaction testing. In summary, our subgroup findings and proposed AIP cutoffs require validation in prospective cohorts with prespecified sample sizes and interaction-testing plans or in randomized trials. Combining AIP with complementary biomarkers such as ApoB/ApoA1 and insulin-resistance indices may strengthen multimarker models to support individualized prevention and treatment strategies.

## 5 Conclusion

An elevated AIP was associated with a higher risk of stroke progression within one year after AIS. This association exhibited a sex-specific threshold effect. A bidirectional mediating relationship between AIP and fibrinogen suggests that they may constitute targets for combined therapeutic intervention. Moreover, interactions of AIP with sex, NIHSS, HbA1c, and CRP provide a rationale for clinical risk stratification, individualized monitoring, and more intensive preventive strategies.

## Data Availability

Publicly available datasets were analyzed in this study. This data can be found at: http://www.Datadryad.org/.

## Footnotes

1
https://doi.org/10.5061/dryad.w0vt4b92c
