# Supplementary material for: Association between atherogenic index of plasma and post-stroke epilepsy within one year in patients with acute ischemic stroke: a retrospective cohort study
Source: Front Neurol. 2026 Mar 30;17:1794488. doi: 10.3389/fneur.2026.1794488 (PMC13070791; doi:10.3389/fneur.2026.1794488)
Supplement: Supplementary file 1 [file Supplementary_File_1.docx]

Supplementary Material

Supplementary Tables:

| **Supplementary Table 1.** Means, SDs, and mean-to-SD ratios of continuous variables. | | | |
| --- | --- | --- | --- |
| **Variable** | **Mean** | **SD** | **Mean/SD** |
| Age (years) | 67.69 | 10.93 | 6.19 |
| NIHSS | 8.05 | 2.96 | 2.72 |
| Platelet count (10^9^/L) | 189.62 | 26.86 | 7.06 |
| White blood cell count (10^9^/L) | 8.40 | 1.49 | 5.64 |
| HbA1c (%) | 6.69 | 0.93 | 7.19 |
| **CRP (mg/L)** | **17.20** | **22.72** | **0.76** |
| Alanine aminotransferase (U/L) | 24.28 | 10.29 | 2.36 |
| Bilirubin (µmol/L) | 15.22 | 4.02 | 3.79 |
| Albumin (g/L) | 40.84 | 2.25 | 18.15 |
| Urea (mmol/L) | 6.47 | 1.41 | 4.59 |
| eGFR (mL/min/1.73m²) | 88.82 | 29.20 | 3.04 |
| Blood uric acid (µmol/L) | 343.15 | 58.62 | 5.85 |
| Fibrinogen (g/L) | 3.61 | 0.47 | 7.68 |
| SD, standard deviation; NIHSS, National Institutes of Health Stroke Scale; HbA1c, Hemoglobin A1c; CRP, C-reactive protein; eGFR, estimated glomerular filtrationrate. | | | |

| **Supplementary Table 2.** Multiplicative and additive interactions between AIP and clinical characteristics on the risk of PSE. | | | | | | |
| --- | --- | --- | --- | --- | --- | --- |
| **Subgroup** | | **AIP** | **OR (95%CI**） | **Multiplicative interaction** | **Additive interaction** | |
|  |  |  |  | ***P*** | **RERI (95%CI)** | **AP (95%CI)** |
| Age (years) | <65 | Q1 | 1(Ref) | 0.690 | -0.81 (-2.14~0.53) | -0.19 (-0.52~0.13) |
|  |  | Q2 | 5.05 (3.40~7.51)* |  |  |  |
|  | ≥65 | Q1 | 0.90 (0.60~1.33) |  |  |  |
|  |  | Q2 | 4.14 (2.75~6.24)* |  |  |  |
| Cortical involvement | No | Q1 | 1(Ref) | 0.820 | 0.27 (-1.68~2.22) | 0.06 (-0.33~0.44) |
|  |  | Q2 | 4.44 (3.45~5.72)* |  |  |  |
|  | Yes | Q1 | 1.18 (0.76~1.85) |  |  |  |
|  |  | Q2 | 4.90 (3.20~7.49)* |  |  |  |
| Adjusted for age, sex, NIHSS, cerebral herniation, hydrocephalus, deep vein thrombosis, diabetes, hypertension, coronary disease, atrial fibrillation, fatty liver, cortical involvement, large vessel disease, platelet count, white blood cell count, HbA1c, CRP, alanine aminotransferase, bilirubin, albumin, urea, eGFR, and blood uric acid. For subgroup analyses conducted within levels of a categorical variable, that variable was not included as a covariate in the subgroup-specific models. **P*<0.001. If the 95% CIs for RERI and AP do not include 0, the interaction between them is considered statistically significant. OR, odds ratio; CI, confidence interval; RERI, relative excess risk due to interaction; AP, attributable proportion; AIP, atherogenic index of plasma; PSE, post-stroke epilepsy; NIHSS, National Institutes of Health Stroke Scale; HbA1c, Hemoglobin A1c; CRP, C-reactive protein; eGFR, estimated glomerular filtrationrate. | | | | | | |

| **Supplementary Table 3.** Association between AIP and PSE in different models. | | | | | | | | |
| --- | --- | --- | --- | --- | --- | --- | --- | --- |
| **Variable** | **Crude** | | **Model 1** | | **Model 2** | | **Model 3** | |
|  | **OR (95%CI)** | ***P*** | **OR (95%CI)** | ***P*** | **OR (95%CI)** | ***P*** | **OR (95%CI)** | ***P*** |
| AIP per 0.1-unit | 1.19 (1.14~1.25) | <0.001 | 1.66 (1.57~1.76) | <0.001 | 1.66 (1.56~1.77) | <0.001 | 2.15 (1.97~2.33) | <0.001 |
| AIP (T1＜0.030) | 1(Ref) |  | 1(Ref) |  | 1(Ref) |  | 1(Ref) |  |
| AIP (T2 0.030~0.135) | 2.01 (1.65~2.46) | <0.001 | 4.89 (3.87~6.17) | <0.001 | 5.07 (4.00~6.44) | <0.001 | 6.77 (5.02~9.13) | <0.001 |
| AIP (T3≥0.135) | 2.66 (2.20~3.23) | <0.001 | 9.10 (7.17~11.54) | <0.001 | 9.29 (7.22~11.95) | <0.001 | 15.33 (10.87~21.61) | <0.001 |
| Model 1: Adjusted for age, sex, and NIHSS. Model 2: Adjusted for age, sex, NIHSS, cerebral herniation, hydrocephalus, deep vein thrombosis, diabetes, hypertension, coronary disease, atrial fibrillation, and fatty liver. Model 3: Adjusted for age, sex, NIHSS, cerebral herniation, hydrocephalus, deep vein thrombosis, diabetes, hypertension, coronary disease, atrial fibrillation, fatty liver, cortical involvement, large vessel disease, platelet count, white blood cell count, HbA1c, CRP, alanine aminotransferase, bilirubin, albumin, urea, eGFR, and blood uric acid. OR, odds ratio; CI, confidence interval; AIP, atherogenic index of plasma; PSE, post-stroke epilepsy; NIHSS, National Institutes of Health Stroke Scale; HbA1c, Hemoglobin A1c; CRP, C-reactive protein; eGFR, estimated glomerular filtrationrate. | | | | | | | | |

Supplementary Figures:


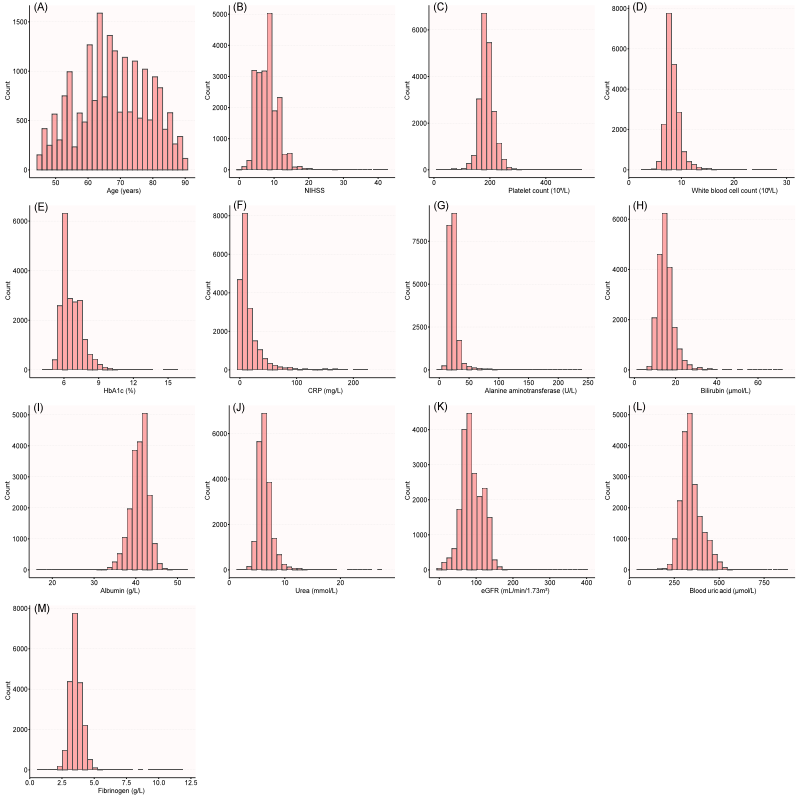


**Supplementary Figure 1.** Histograms of continuous variables. NIHSS, National Institutes of Health Stroke Scale; HbA1c, Hemoglobin A1c; CRP, C-reactive protein; eGFR, estimated glomerular filtrationrate.


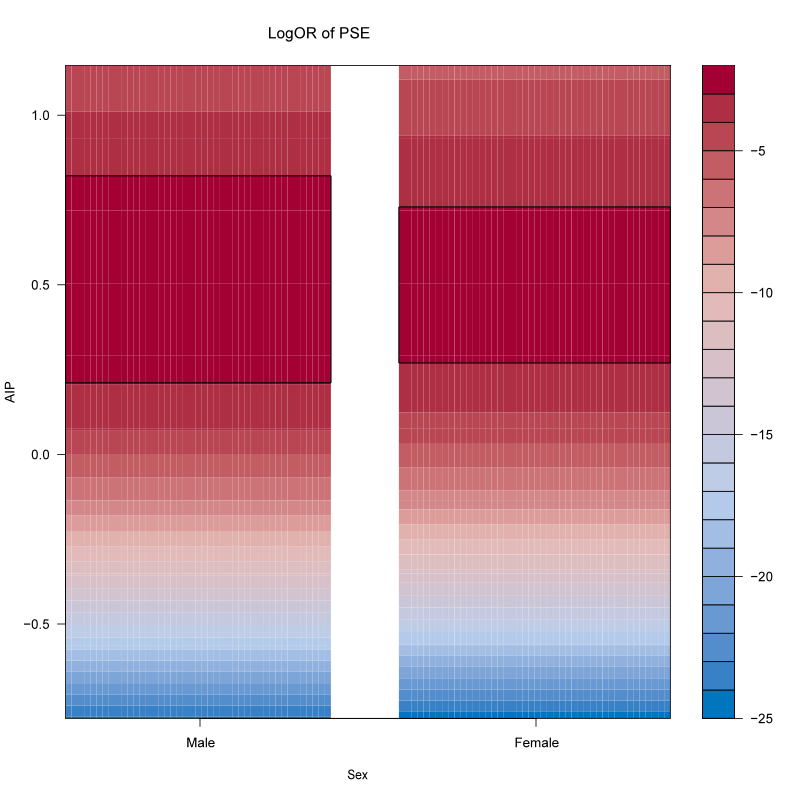


**Supplementary Figure 2.** Two-dimensional heat map visualization between AIP and PSE among sex. Adjusted for age, NIHSS, cerebral herniation, hydrocephalus, deep vein thrombosis, diabetes, hypertension, coronary disease, atrial fibrillation, fatty liver, cortical involvement, large vessel disease, platelet count, white blood cell count, HbA1c, CRP, alanine aminotransferase, bilirubin, albumin, urea, eGFR, and blood uric acid. OR, odds ratio; AIP, atherogenic index of plasma; PSE, post-stroke epilepsy; NIHSS, National Institutes of Health Stroke Scale; HbA1c, Hemoglobin A1c; CRP, C-reactive protein; eGFR, estimated glomerular filtrationrate.
